# Orchids at the Intersection of Climate, Biotic Interactions, and Evolutionary Dynamics

**DOI:** 10.3390/plants15111754

**Published:** 2026-06-05

**Authors:** Zuzana Štípková, Vladan Djordjević

**Affiliations:** 1 Department of Biodiversity Research, Global Change Research Institute CAS, 603 00 Brno, Czech Republic; 2 Institute of Botany and Botanical Garden, Faculty of Biology, University of Belgrade, Takovska 43, 11000 Belgrade, Serbia

Orchids (Orchidaceae) are among the most species-rich and ecologically specialised families of flowering plants, combining remarkable evolutionary success with exceptional sensitivity to environmental changes. Their persistence depends not only on climatic conditions but also on highly specific interactions with pollinators, mycorrhizal fungi, soil characteristics, vegetation types, and other microhabitat features. The contributions collected in this Special Issue, Orchid Conservation and Biodiversity, collectively emphasise the need for integrative approaches that bridge abiotic drivers, biotic interactions, and evolutionary processes to fully understand orchid ecology, biogeography, and diversification.

Taken together, the eight studies included in this Special Issue offer complementary perspectives on how orchid distributions and diversity patterns result from interacting constraints across spatial and temporal scales Contributions 1–8. Several contributions highlight the central role of climate, particularly precipitation-related variables, in defining the broad environmental limits for orchid occurrence Contributions 1,2. These studies confirm that moisture availability is critical for key life-history stages, influencing germination success, mycorrhizal activity, and flowering dynamics. However, an important and recurring conclusion is that climatic suitability alone rarely explains actual orchid distributions.

A major advance from this collection is the explicit consideration of biotic interactions as limiting factors. Studies incorporating pollinator distributions show that even climatically suitable habitats may remain unoccupied when pollinator availability is limited in space and/or time Contributions 3,4. This is particularly evident in orchids with specialised pollination systems, including mimicry-based interactions, where successful reproduction depends on the presence of specific insect taxa. These findings reinforce the idea that pollinators are critical determinants of orchid range limits and highlight the insufficiency of climate-only predictive models.

Beyond contemporary ecological patterns, several articles in this Special Issue place orchid diversity within an evolutionary and historical framework Contributions 5–7. Orchids are often associated with fragmented, unstable, or transient habitats, where populations experience repeated bottlenecks, developments, and local extinctions. These demographic conditions can amplify the effects of genetic drift while also facilitating rapid divergence when combined with strong reproductive isolation mechanisms. The interaction between habitat instability and specialised pollination systems emerges as a powerful driver of diversification, potentially enabling speciation even without prolonged geographic isolation.

Detailed morphological, molecular, and plastomic analyses were conducted on *Cypripedium × ventricosum* Sw. from Changbai Mountain, China Contribution 5. This study clarified the relationships among *Cypripedium* taxa with similar morphological characteristics, providing a foundation for research on orchid evolution and species conservation Contribution 5. A special study conducted multi-stage comparative analyses of purple- and white-flowered *Arundina graminifolia* across bud, post-bud, and blooming stages Contribution 7. This study establishes that insertional mutagenesis in the *AgDFR* coding region underlies the natural white flower in *A. graminifolia* Contribution 7. The developed molecular marker allows reliable differentiation of white-flowered variants from pigmented counterparts, providing valuable tools for germplasm management and breeding programmes Contribution 7. In addition, phylogenetic analysis, conserved protein domain analysis, gene structure analysis, *cis*-element analysis, and expression pattern analysis of the SPL gene family in *Phalaenopsis equestris* were performed as part of a special study Contribution 8.

Pollination biology occupies a central position in this evolutionary context. Highly specific pollinator relationships can severely limit gene flow among populations, reinforcing divergence initiated by ecological or geographic factors Contributions 6,7. Although such specialisation may reduce reproductive output at the population level, it may also enhance lineage persistence by promoting adaptive differentiation in heterogeneous landscapes. The studies included here highlight that pollination strategies should be viewed not only as ecological traits, but also as key evolutionary mechanisms shaping orchid diversification.

Several contributions highlight the importance of fine-scale habitat heterogeneity and microenvironmental constraints Contributions 2,8. Local variation in substrate properties, host availability, or associated fungal communities can strongly influence orchid establishment and survival, often at spatial scales not represented by regional environmental datasets. These findings underscore the need to integrate microhabitat-level data with broader-scale models to assess orchid ecological requirements and vulnerability more accurately.

Overall, this Special Issue demonstrates that orchid distributions, persistence, and diversification are best understood as emergent outcomes of interacting abiotic conditions, biotic dependencies, and evolutionary histories Contributions 1–8. Climate sets broad physiological boundaries, but the availability of suitable habitats depends critically on functional ecological networks involving pollinators and symbiotic partners. Over evolutionary timescales, repeated environmental changes and habitat turnover further amplify the consequences of these interactions, shaping both diversity patterns and extinction risk.

From a conservation perspective, the collective insights presented here have important implications. Predictive frameworks and management strategies that focus solely on climatic suitability may substantially overestimate future orchid persistence if biotic partners do not track shifting environments. Effective orchid conservation therefore requires network-based and process-oriented approaches, emphasising habitat connectivity, maintenance of biotic interactions, and protection of ecological dynamics alongside species-level targets Contributions 3,4,8.

In conclusion, the studies brought together in this Special Issue highlight the unique value of orchids as model systems for investigating how biodiversity is generated and maintained under environmental complexity and change. By integrating ecological modelling, biotic interactions, and evolutionary perspectives, this collection provides a more comprehensive understanding of orchid biology and offers a solid foundation for future research aimed at conserving one of the most remarkable plant families.

